# Factors associated with sports-related dental injuries among young athletes: a cross-sectional study in Miyagi prefecture

**DOI:** 10.1186/s12903-017-0466-2

**Published:** 2017-12-29

**Authors:** Shinobu Tsuchiya, Masahiro Tsuchiya, Haruki Momma, Takuya Sekiguchi, Kaoru Kuroki, Kenji Kanazawa, Takeyoshi Koseki, Kaoru Igarashi, Ryoichi Nagatomi, Yoshihiro Hagiwara

**Affiliations:** 1 Division of Oral Dysfunction Science, Tohoku University Graduate School of Dentistry, Sendai, 980–8575 Japan; 2 Department of Nursing, Tohoku Fukushi University, Sendai, 981–8522 Japan; 3 Department of Medicine and Science in Sports and Exercise, Tohoku University Graduate School of Medicine, Sendai, 980–8574 Japan; 4 Division of Biomedical Engineering for Health & Welfare, Tohoku University Graduate School of Biomedical Engineering, Sendai, 980–8574 Japan; 5 Department of Orthopaedic Surgery, Tohoku University Graduate School of Medicine, Sendai, 980–8574 Japan; 6 Department of Rehabilitation, Tohoku Fukushi University, Sendai, 981–8522 Japan; 7 Division of Preventive Dentistry, Tohoku University Graduate School of Dentistry, Sendai, 980–8575 Japan

**Keywords:** Sports injuries, Traumatic dental injuries, Young male athletes, Coaching, Physical/psychological distress, Cross-sectional study

## Abstract

**Background:**

Sports-related dental injuries, such as tooth fracture, loosening, and avulsion, are a major concern among young athletes because they directly impair oral function. Although the preventive efficacy of mouthguards has been well established, the prevalence of sports-related dental injuries remains high among young athletes. The aim of this study is to identify the variables contributing to the risk of sports-related dental injuries by conducting a survey on large population of young athletes in Miyagi prefecture.

**Methods:**

A cross-sectional study was conducted with school-aged athletes (aged 6–15 years, *n* = 5735) using a self-reported questionnaire. The questionnaire examined general variables, including sex, age, and body mass index; sports-related variables, including sports-type, team level, activity schedule, break time, and verbal/physical abuse by coaches; and lifestyle variables related to free time, including screen-time and sleep duration. Their associations with sports-related dental injuries were examined using multivariate logistic regression models.

**Results:**

The prevalence of sports-related dental injuries was 13.3% (763 of 5735 young athletes) and was higher in males (14.3%, 592 of 4132) than in females (10.7%, 171 of 1603; adjusted odds ratios [ORs] and 95% confidence intervals [CIs]: 1.48 [1.22–1.79], *p* < 0.001). After stratification according to sex, significant associations with the prevalence of sports-related dental injuries were evident for three variables—insufficient break time, verbal abuse, and physical punishment—in males (adjusted ORs [95% CI]: 1.35 [1.03–1.77], *p* = 0.032; 1.31 [1.05–1.62], *p* = 0.015; and 1.36 [1.06–1.75], *p* = 0.016, respectively) but not in females (adjusted ORs [95% CI]: 0.88 [0.53–1.47], *p* = 0.623; 1.29 [0.87–1.91], *p* = 0.206; and 0.97 [0.57–1.63], *p* = 0.894, respectively).

**Conclusions:**

Although our results might be based on the individual athlete’s self-perception to the sports-related variables, our results suggest that insufficient break time, verbal abuse, and physical punishment from coaches are positively associated with the prevalence of sports-related dental injuries in young male athletes.

## Background

Traumatic dental injuries are a major oral health problem in children and adolescents [1–3]. Severe dental trauma to the teeth and/or periodontium, such as tooth fracture, loosening, and avulsion [4, 5], directly results in the impairment of oral functions such as chewing and speech [6, 7]. In addition to physical aspects, it also affects psychosocial development through aesthetic concerns [8]. As reported in a recent meta-analysis, the prevalence of dental injuries is 17.5% in children and adolescents worldwide and two times higher in boys than that in girls [9]. Because approximately 40% of all dental injuries occur during sports activities, the annual treatment costs for sports-related dental injuries were reportedly more than US $2 million per 1 million inhabitants [2, 10–13]. Thus, the identification of factors associated with the prevalence of sports-related dental injuries in children is an important step toward their prevention.

Sports experts including dentists recommend that mouthguards be worn to prevent sports-related dental injuries. Mouthguards markedly reduce the incidence of sports-related dental injuries among athletes, especially those playing full-contact sports such as rugby and football [12, 14–17]. However, because the wearing of mouthguards is not mandatory for young athletes participating in sports with less contact [12, 17, 18], the prevalence of sports-related dental injuries remains high [11, 12, 14–18]. Furthermore, tooth eruption and jaw growth in mixed dentition can mean that the fit of mouthguards is impermanent and will tend to be worn less often by young athletes, as a result [12, 16–18]. Thus, the use of mouthguards alone is insufficient for the prevention of sports-related dental injuries in young athletes.

Recently, intrinsic (e.g., age, sex, body mass index [BMI], physical ability, fatigue, playing career, sleeping behavior, and mental condition) and extrinsic risk factors, especially related to the playing environment (e.g., contact sports, coaching, safety equipment, and team management) for sports injuries have been recognized for the protection of athletes’ health [19–25]. Regarding the prevalence of sports-related dental injuries, although it has been determined that several risk factors including sex, BMI, and malocclusion (e.g., large overjet) are positively associated with the prevalence [1, 11, 26], playing environmental factors, excluding the use of mouthguards, have not been examined sufficiently. In particular, because young athletes are mostly playing under the management of sports teams and coaches [20, 27, 28], that the playing environmental factors such as coaching and activity load might have an impact on the risk of sports-related dental injuries.

Understanding the causes of sports-related dental injuries will enable the development of effective strategies for their prevention and advance the oral health of future athletes [11, 20, 22, 24, 29]. In this study, we investigated factors associated with sports-related dental injuries by a cross-sectional study using a large population sample of young Japanese athletes.

## Methods

### Participants

**Fig. 1:**
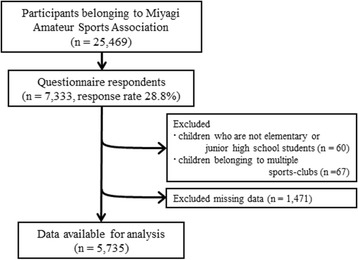
Flowchart of participant recruitment

The methods complied with the Strengthening the Reporting of Observational Studies in Epidemiology (STROBE) statement [30]. The participants were young athletes aged 4–18 years belonging to the Miyagi Amateur Sports Association, which was established in Miyagi prefecture with the aim of promoting the health of young people through sports (baseball, soccer, basketball, volleyball, judo, kendo, karate, athletics, badminton, swimming, etc.). A self-administered questionnaire and document about informed consent were mailed to the 25,469 members in October 2014. By the end of December 2014, 7333 had replied with both written informed consent and the completed questionnaire (a response rate of 28.8%) [23, 31]. If children under the age of 16 needed to participate, the parent or guardian of the child also provided informed consent on behalf of the child. Additionally, those who belonged to multiple sports clubs (*n* = 67) were excluded from this study. Those with missing data on potential covariates (*n* = 1471) were also excluded. The final study population comprised 5735 children (4132 males and 1603 females; Fig. 1). The study protocol was reviewed and approved by the Ethics Committee at the Tohoku University Graduate School of Medicine, Sendai, Japan (approval number: 2013–1-564).

### Questionnaire

The design of the questionnaire has been previously described [23, 31]. Briefly, the questionnaire gathered data on general information (sex, age, educational stage, height, and weight), sports-related information (sports type, team level, number of years spent playing the sport, activity schedule, enjoyment of playing, break time during practices, and verbal abuse or physical punishment by coaches), and information on other habitual daily activities (screen time spent playing games or watching television and sleep duration).

The prevalence of sports-related dental injuries was assessed by the aforementioned self-reported questionnaire. The question, in a query series related to sports injuries, was expressed thus: “Have you ever experienced a dental injury such as tooth fracture, loosening, or avulsion?” Answers were “yes” or “no” [26, 32, 33], which were categorized into two groups, injured (“yes”) and non-injured (“no”).

BMI was categorized into three groups (underweight: <18.5, normal weight: 18.5 to <25, and overweight: ≥25). The other following continuous variables were divided into categories according to their distribution: number of years spent playing sports was categorized into three groups (≤1, >1 to <5, and ≥5 years); number of days per week participating in team practice or games was categorized into two groups (<3 and ≥3 days); average hours of screen time per day was categorized into three groups (≤3, >3 to <5, and ≥5 h); and average hours of sleep duration per day was categorized into three groups (≤8.5, >8.5 to <9, and ≥9 h).

Sports type was categorized into four groups according to the degree of contact (non-contact sports: tennis, athletics, table tennis, and badminton; limited-contact sports: baseball, and volleyball; semi-contact sports: kendo and karate; and full-contact sports: soccer, basketball, judo, and handball) [34]. It should be noted that rubber-ball baseball, baseball, and softball were categorized as baseball; mini-basketball and basketball as basketball; and tennis and soft tennis as tennis. Precoded answers for team level were “national competition,” “district competition in Tohoku region,” “prefectural competition,” “area competition,” and “recreation alone.” These levels were categorized into three groups: (1) area competition (local competition or recreation alone), (2) prefectural competition, and (3) regional competition (district competition in the Tohoku region or national competition). Enjoyment in playing was categorized into two groups (yes [“Strongly agree” and “Somewhat agree”] and no [“Somewhat disagree,” “Strongly disagree,” and “difficult to say”]), and break time during practices was categorized into two groups (sufficient [“often” and “just enough”] and insufficient [“less than enough”]).

The following questions were also used to assess the verbal abuse from coaches: “Have you ever felt your coach to be verbally offensive against you in the past year?” and “Have you ever felt your coach to be verbally offensive against any member of your team in the past year?”; and the physical punishment from coaches: “Did your coach slap or kick you in the past year?” and “Did your coach slap or kick any of the members of your team in the past year?”. Participants answered these questions “yes” or “no.” These were categorized into two groups according to the answers to the questions as follows: (absence: “no” response to the questions on verbal abuse and physical punishment; presence: “yes” response to either of the questions on verbal abuse or physical punishment).

### Statistical analysis

Data were presented as numbers and percentages (%). Multiple logistic regression analysis was used to examine the association between the prevalence of sports-related dental injuries and other variables, using the experience of sports-related dental injuries as an objective and other variables as explanatory variables. The effect of multicollinearity for all variables was assessed using variance inflation factors (VIFs), the highest value of which was 1.412 for the educational stage in female athletes, suggesting that the collinearity of the study variables was not a significant problem [35]. Odds ratios (ORs) and 95% confidence intervals (CIs) were calculated for the prevalence of sports-related dental injuries. These analyses were performed using the forced-entry method for all variables after stratification by sex because of distinct sex differences. All statistical analyses were performed using SPSS version 23.0 (SPSS Japan Inc., Tokyo, Japan). All tests were two-tailed, and *p* < 0.05 was considered statistically significant.

## Results

The characteristics of the participants are shown in Table 1. The median age of participants was 11 years (interquartile range [IQR]: 10–12 years). Their median height and weight were 145 cm (IQR: 135–154 cm) and 36 kg (IQR: 30.0–44.9 kg), respectively. Seven hundred sixty-three (13.3%) of 5735 participants reported the experience of sports-related dental injuries, and the prevalence of all sports injuries was 46.2% (1893 of 4132 male participants, and 757 of 1603 female participants). After stratification by sex (Table 2), sports-related dental injuries were reported by 14.3% (592 of 4132) of male participants and 10.7% (171 of 1603) of female participants. The prevalence of sports-related dental injuries in male participants was higher than that in female participants for each category of contact sports. When analyzing the total data set by multiple logistic regression analysis and including sex as an independent variable, the OR (95% CI) for the prevalence of sports-related dental injuries in male participants compared with that in female participants was 1.48 (1.22–1.79, *p* < 0.001). Indeed, it should be noted that there were no significant multiplicative interactions between sex and other variables by the Wald test.

**Table 1:** Characteristics of participants with or without dental injury

Variables		Dental injury		
		Non-injured, *n* (%)	Injured, *n* (%)	
	Total	4972 (86.7)	763 (13.3)	*p* value
Sex	Males	3540 (85.7)	592 (14.3)	**<0.001**
	Females	1432 (89.3)	171 (10.7)	
Grade	Elementary school	3926 (86.7)	604 (13.3)	0.900
	Junior high school	1046 (86.8)	159 (13.2)	
BMI	Underweight	3327 (86.2)	532 (13.8)	0.238
	Normal weight	1508 (87.5)	215 (12.5)	
	Overweight	137 (89.5)	16 (10.5)	
Sport	Baseball	1333 (86.8)	202 (13.2)	0.989
	Soccer	1051 (86.4)	166 (13.6)	
	Volleyball	464 (87.1)	69 (12.9)	
	Basketball	1083 (87.1)	161 (12.9)	
	Judo	175 (85.8)	29 (14.2)	
	Kendo	339 (85.6)	57 (14.4)	
	Karate	160 (87.4)	23 (12.6)	
	Handball	28 (82.4)	6 (17.6)	
	Tennis	96 (85.0)	17 (15.0)	
	Athletics	97 (85.8)	16 (14.2)	
	Table Tennis	57 (89.1)	7 (10.9)	
	Badminton	89 (89.9)	10 (10.1)	
Contact sport	Non-contact	339 (87.1)	50 (12.9)	0.960
	Limited-contact	2868 (87.0)	430 (13.0)	
	Semi-contact	508 (86.2)	81 (13.8)	
	Full-contact	1257 (86.2)	202 (13.8)	
Team level	Local competition	2844 (87.2)	416 (12.8)	**0.019**
	Prefectural competition	1752 (86.8)	267 (13.2)	
	District competition	376 (82.5)	80 (17.5)	
Experience (years)	≤1	2138 (87.4)	309 (12.6)	0.428
	>1 to <5	2269 (86.2)	363 (13.8)	
	≥5	565 (86.1)	91 (13.9)	
Training days per week	<3	2846 (87.2)	417 (12.8)	0.179
	≥3	2126 (86.0)	346 (14.0)	
Enjoyment in playing sport	Yes	4880 (86.8)	739 (13.2)	**0.018**
	No	92 (79.3)	24 (20.7)	
The time for breaks	Sufficient	4453 (87.1)	662 (12.9)	**0.007**
	Insufficient	499 (83.2)	101 (16.8)	
Verbal abuse from coaches	No	3954 (87.6)	559 (12.4)	**<0.001**
	Yes	1018 (83.3)	204 (16.7)	
Physical punishment from coaches	No	4388 (87.4)	634 (12.6)	**<0.001**
	Yes	584 (81.9)	129 (18.1)	
Screen time per day (hours)	≤3	1772 (86.7)	273 (13.3)	0.168
	>3 to <5	1836 (87.5)	263 (12.5)	
	≥5	1364 (85.7)	227 (14.3)	
Sleep time per day (hours)	≤8.5	1725 (85.9)	284 (14.1)	0.384
	>8.5 to <9	1009 (87.0)	151 (13.0)	
	≥9	2238 (87.2)	328 (12.8)	

*BMI* body mass index

*P* values representing significant differences (<0.05) are indicated in bold

**Table 2:** Characteristics of participants with or without dental injuries according to sex

Variables	Category	Males			Females		
		Non-injured, *n* (%)	Injured, *n* (%)	*p* value	Non-injured, *n* (%)	Injured, *n* (%)	*p* value
		3540 (85.7)	592 (14.3)		1432 (89.3)	171 (10.7)	
Grade	Elementary school	2909 (85.9)	478 (14.1)	0.402	1017 (89.0)	126 (11.0)	0.467
	Junior high school	631 (84.7)	114 (15.3)		415 (90.2)	45 (9.8)	
BMI	Underweight	2391 (85.1)	418 (14.9)	0.199	936 (89.1)	114 (10.9)	0.908
	Normal weight	1034 (86.5)	161 (13.5)		474 (89.8)	54 (10.2)	
	Overweight	115 (89.8)	13 (10.2)		22 (88.0)	3 (12.0)	
Sports	Baseball	1260 (86.5)	196 (13.5)		73 (92.2)	5 (7.8)	
	Soccer	992 (86.0)	161 (14.0)		59 (92.2)	5 (7.8)	
	Volleyball	115 (85.8)	19 (14.2)		349 (87.5)	50 (12.5)	
	Basketball	582 (85.2)	101 (14.8)		501 (89.3)	60 (10.7)	
	Judo	123 (82.6)	26 (17.4)		52 (94.5)	3 (5.5)	
	Kendo	215 (84.3)	40 (15.7)		124 (87.9)	17 (12.1)	
	Karate	117 (86.0)	19 (14.0)		43 (91.5)	4 (8.5)	
	Handball	15 (75.0)	5 (25.0)		13 (92.9)	1 (7.1)	
	Tennis	38 (80.9)	9 (19.1)		58 (87.9)	8 (12.1)	
	Athletics	38 (84.4)	7 (15.6)		59 (86.8)	9 (13.2)	
	Table Tennis	20 (80.0)	5 (20.0)		37 (94.9)	2 (5.1)	
	Badminton	25 (86.2)	4 (13.8)		64 (91.4)	6 (8.6)	
Contact sport	Non-contact	121 (82.9)	25 (17.1)	0.554	218 (89.7)	25 (10.3)	0.796
	Limited-contact	1948 (86.1)	314 (13.9)		920 (88.8)	116 (11.2)	
	Semi-contact	339 (85.0)	60 (15.0)		169 (89.9)	21 (11.1)	
	Full-contact	1132 (85.4)	193 (14.6)		125 (93.3)	9 (6.7)	
Team level	Area competition	2059 (86.3)	328 (13.7)	**0.026**	785 (89.9)	88 (10.1)	0.252
	Prefectural competition	1262 (85.7)	210 (14.3)		490 (89.6)	57 (10.4)	
	Regional competition	219 (80.2)	54 (19.8)		157 (85.8)	26 (14.2)	
Experience (years)	≤1	1481 (86.2)	237 (13.8)	0.540	657 (90.1)	72 (9.9)	0.280
	1 to 5	1653 (85.6)	279 (14.4)		616 (88.0)	84 (12.0)	
	≥5	406 (84.2)	76 (15.8)		159 (91.4)	15 (8.6)	
Training days per week (days)	<3	2185 (86.6)	337 (13.4)	**0.027**	661 (89.2)	80 (10.8)	0.877
	≥3	1355 (84.2)	255 (15.8)		771 (89.4)	91 (10.6)	
Enjoyment in playing sport	Yes	3484 (85.8)	575 (14.2)	**0.027**	1396 (89.5)	164 (10.5)	0.227
	No	56 (76.7)	17 (23.3)		36 (83.7)	7 (16.3)	
The time for breaks	Sufficient	3212 (86.3)	511 (13.7)	**0.001**	1261 (89.3)	151 (10.7)	0.925
	Insufficient	328 (80.2)	81 (19.8)		171 (89.5)	20 (10.5)	
Verbal abuse from coaches	No	2841 (86.7)	434 (13.3)	**<0.001**	1113 (89.9)	125 (10.1)	0.173
	Yes	699 (81.6)	158 (18.4)		319 (87.4)	46 (12.6)	
Physical punishment from coaches	No	3116 (86.5)	485 (13.5)	**<0.001**	1272 (89.5)	149 (10.5)	0.510
	Yes	424 (79.8)	107 (20.2)		160 (87.9)	22 (12.1)	
Screen time per day (hours)	<3	1189 (85.8)	197 (14.2)	0.426	583 (88.5)	76 (11.5)	0.231
	3 to 5	1332 (86.2)	213 (13.8)		504 (91.0)	50 (9.0)	
	≥5	1019 (84.8)	182 (15.2)		345 (88.5)	45 (11.5)	
Sleep time per day (hours)	≤8.5	1126 (84.4)	208 (15.6)	0.277	599 (88.7)	76 (11.3)	0.723
	8.5 to 9	729 (86.3)	116 (13.7)		280 (88.9)	35 (11.1)	
	≥9	1685 (86.3)	268 (13.7)		553 (90.2)	60 (9.8)	

*BMI* body mass index

P values representing significant differences (<0.05) are indicated in bold

The adjusted ORs (95% CI) for the prevalence of sports-related dental injuries among the independent variables are shown in Table 3. Insufficient break time during practices and both verbal abuse and physical punishment by the coach were positively associated with the prevalence of sports-related dental injuries in males (adjusted ORs [95% CI]: 1.35 [1.03–1.77], *p* = 0.032; 1.31 [1.05–1.62], *p* = 0.015; and 1.36 [1.06–1.75], *p* = 0.016, respectively) but not in females (adjusted ORs [95% CI]: 0.88 [0.53–1.47], *p* = 0.623; 1.29 [0.87–1.91], *p* = 0.206; and 0.97 [0.57–1.63], *p* = 0.894, respectively). Although there was no significant association between the other variables and the prevalence of sports-related dental injuries after adjusting for covariates, the participants with the highest team level (restricted regional and/or national competitions) had a higher tendency to experience sports-related dental injuries than those at a lower team level (adjusted ORs [95% CI]: 1.35 [0.96–1.90], *p* = 0.085 in males and 1.55 [0.93–2.58], *p* = 0.091 in females).

**Table 3:** Adjusted odds ratios (ORs) for the prevalence of dental injuries

Variables	Category	Males		Females	
		Adjusted OR (95% CI)	*p* value	Adjusted OR (95% CI)	*p* value
Grade	Elementary school	1.00		1.00	
	Junior high school	1.03 (0.79–1.33)	0.856	0.87 (0.55–1.37)	0.544
BMI	Underweight	1.00		1.00	
	Normal weight	0.85 (0.69–1.04)	0.119	0.97 (0.67–1.41)	0.865
	Overweight	0.64 (0.35–1.15)	0.134	1.25 (0.36–4.40)	0.729
Contact sport	Non-contact	1.00		1.00	
	Limited-contact	0.72 (0.45–1.15)	0.167	1.16 (0.67–2.00)	0.594
	Semi-contact	0.76 (0.45–1.29)	0.311	1.07 (0.57–2.03)	0.830
	Full-contact	0.77 (0.48–1.21)	0.257	0.95 (0.57–1.59)	0.841
Team level	Local competition	1.00		1.00	
	Prefectural competition	0.99 (0.81–1.19)	0.875	1.04 (0.72–1.51)	0.818
	District competition	1.35 (0.96–1.90)	0.085	1.55 (0.93–2.58)	0.091
Experience (years)	≤1	1.00		1.00	
	>1 to <5	1.05 (0.86–1.27)	0.642	1.19 (0.85–1.68)	0.315
	≥5	1.14 (0.85–1.53)	0.387	0.74 (0.40–1.39)	0.349
Training days per week (days)	<3	1.00		1.00	
	≥3	1.15 (0.96–1.39)	0.137	0.94 (0.66–1.34)	0.740
Enjoyment in playing sport	Yes	1.00		1.00	
	No	1.44 (0.81–2.54)	0.214	1.62 (0.69–3.84)	0.270
The time for breaks	Sufficient	1.00		1.00	
	Insufficient	1.35 (1.03–1.77)	**0.032**	0.88 (0.53–1.47)	0.623
Verbal abuse from coaches	No	1.00		1.00	
	Yes	1.31 (1.05–1.62)	**0.015**	1.29 (0.87–1.91)	0.206
Physical punishment from coaches	No	1.00		1.00	
	Yes	1.36 (1.06–1.75)	**0.016**	0.97 (0.57–1.63)	0.894
Screen time per day (hours)	≤3	1.00		1.00	
	>3 to <5	0.91 (0.74–1.13)	0.399	0.73 (0.50–1.07)	0.107
	≥5	1.02 (0.81–1.28)	0.866	0.98 (0.65–1.48)	0.920
Sleep time per day (hours)	≤8.5	1.00		1.00	
	>8.5 to <9	0.87 (0.69–1.10)	0.241	0.89 (0.57–1.34)	0.520
	≥9	0.87 (0.69–1.10)	0.246	0.76 (0.50–1.16)	0.206

*Adjusted for the grade, body mass index (BMI), contact sport, team level, years of athletic experience, training days per week, enjoyment in playing, time for breaks, verbal abuse from coaches in the team, physical punishment from coaches in the team, screen time per day, sleep time per day and presence of dental injury. P values representing significant differences (<0.05) are indicated in bold

## Discussion

The cross-sectional study of young Japanese athletes revealed that the prevalence of sports-related dental injuries strongly varies according to sex. Moreover, as our crucial findings, three variables —insufficient break time, and verbal abuse/physical punishment from coaches—were positively associated with a higher prevalence of sports-related dental injuries only in male participants but not in female participants. Although these results might mostly be based on the self-perceptions of the participant athletes, this is the first report to examine the association between management of the athletic environment and sports-related dental injuries.

Few studies have explored the prevalence of sports-related dental injuries in young athletes. Although in previous reports the prevalence of sports-related dental injuries for various sports has ranged from 8.5% to 61.3% [9, 10, 12, 16, 18], our survey determined that the prevalence to be 13.3% (i.e., approximately one-eighth of young Japanese athletes have experienced sports-related dental injuries). Similar to previous reports [1–3, 10], our results indicated a significantly higher prevalence (1.5 times) of sports-related dental injuries in male athletes than in female athletes. Sex differences in sports injuries may be caused by a complex mix of intrinsic (e.g., biological differences, physical strength, weight, and psychological characteristics) and playing environmental factors (e.g., playing management including coaching styles) [19, 21, 23, 34, 35]. Young female athletes have higher rates of sports dropout for reasons including the unfavorable coaching behavior (e.g., autocratic control/lower autonomy) [36, 37], while previous studies of athletes’ preferences for coaching leadership behaviors concluded that male athletes preferred a more autocratic coaching style, such as more intensive training [38, 39]. Altogether, our findings underscore that sports-related dental injuries are characteristically male-dominant, which may be based on sex differences in susceptibility to coaching styles.

Unlike sports injuries, which include chronic overuse and damage accumulation, most sports-related dental injuries are caused by accidental direct impacts [2, 10, 11, 19, 24]. Therefore, no significant associations between sports-related dental injuries and most common risk factors in sports injuries (e.g., BMI, contact sports, playing career, and sleep disruption) could be found in this study. However, as with sports injuries [20, 22, 23, 25, 40, 41], the playing environmental factors, including self-perceived coaching styles, are positively associated with the self-perceived prevalence of sports-related dental injuries. Male participants who felt that break time was insufficient during sports practice show a higher prevalence of sports-related dental injuries, but there was no association with the frequency of sports activity. In addition, the self-perceived physical punishment or verbal abuse from coaches of young male athletes was positively associated with a higher prevalence of sports-related dental injuries. Unlike with physical punishment, verbal abuse should include less direct physical distress, but remarkably it also was associated with the self-perceived prevalence of sports-related dental injuries. These findings suggest that the incidence of sports-related dental injuries not only depends on an excessive workload but also depends on psychological distress. Since the International Olympic Committee warns that young athletes have a risk to face not only the violence [22, 29] but also negative influences [27, 29, 42, 43], sports experts must mention whether abused athletes play with aggression and inflict sports injuries on other teammates as part of a “negative chain reaction” caused by the abuse [29, 42]. Thus, the prevalence of sports-related dental injuries among young athletes, especially males, must be acknowledged to be associated not only with an excessive workload but also with psychological stress.

This study had some limitations. First, the survey revealed low response rates (28.8%), possibly owing to less follow-up strategy. The generalizability of our findings may be limited because we cannot rule out the possibility of a selection bias [44]. Meanwhile, because victims of abuse often avoid publicizing this fact, a higher prevalence of physical and/or psychologic abuse may exist among the nonrespondents [45, 46]. Thus, our results could have been clearer if the nonrespondents had contributed. Second, the experience of sports-related dental injuries depends upon participants’ memory and self-perception. A standardized dental examination and/or checking medical care costs covered by sports injury insurance services are good ways to survey the prevalence of sports-related dental injuries [6, 10, 12, 16, 18]. After a dental examination, the final judgment on whether traumatic dental injuries were sports-related should be made based on the participant’s memory [12, 18]; the use of insurance records carries the risk of excluding mild incidents of sports-related dental injuries that were not claimed for [10]. Third, our questionnaire did not include a query about the use of mouthguards. Although mouthguards markedly reduce the incidence of sports-related dental injuries among athletes [12, 14–17], the use of mouthguards, especially among young athletes, probably depends on the coaching quality. Since the prevalence of sports-related dental injuries remains high (more than one-eighth of young athletes), there must be other strategies for the prevention of sports-related dental injuries. In summary, a future survey with a more appropriate design may reveal the underlying relations between sports-related dental injuries and potential risk factors shown in this study.

## Conclusions

Our study is the first to show three variables (i.e., insufficient break time, verbal abuse from coaches, and physical punishment from coaches) associated with the self-perceived prevalence of sports-related dental injuries among young athletes.

## Abbreviations

BMI: Body mass index
CI: Confidence interval
OR: Odds ratio
